# What do European clinical guidelines say about genetic testing for people with neuropsychiatric disorders? A scoping review

**DOI:** 10.1097/YPG.0000000000000407

**Published:** 2025-12-03

**Authors:** Izemnur Arican, Marte van der Horst, Nicholas Bass, Janneke R. Zinkstok

**Affiliations:** a Mental Health Neuroscience Department, Division of Psychiatry, University College London; b East London NHS Foundation Trust, London, UK; c Department of Psychiatry, Brain Center, University Medical Center Utrecht, Utrecht; d Department of Psychiatry, Radboud University Medical Center, Donders Institute for Brain, Cognition, and Behavior; e Karakter Child and Adolescent Mental Health Care, Nijmegen, The Netherlands

**Keywords:** genetic, guideline, neuropsychiatric disorders, review, scoping, testing

## Abstract

Genomic medicine has progressed rapidly, and many high-risk genetic variants for neuropsychiatric disorders have now been identified. However, clinical genetic testing is rarely utilized in psychiatric settings. This scoping review examined European clinical practice guidelines (CPGs) for genetic testing in neuropsychiatric disorders to map recommendations and identify gaps. Seventeen CPGs published since 2010 met the inclusion criteria. There was a wide variation in scope, quality, and conditions covered. Seven CPGs addressed autism spectrum disorder, generally recommending testing when additional features such as intellectual disability or dysmorphology were present. One CPG covered the investigation of intellectual disability, advising Fragile X testing, chromosomal microarray, and whole-genome sequencing. Most CPGs (11/17), related to dementia, advising testing with very early onset and/or an indicative family history. Overall, European CPGs for genetic testing in psychiatry vary significantly, contributing to clinician uncertainty. Harmonizing evidence-based CPGs is crucial to advance the integration of genetic testing in psychiatric practice.

## Introduction

A genome-wide approach can now be taken to genetic investigation in the clinic. This has been made possible by technological advances, such as genome-wide genotyping microarray and whole genome sequencing (WGS). Of course, most neuropsychiatric disorders have a complex etiology: with both genetic and nongenetic factors contributing to the development of the illness (Sullivan and Geschwind, 2019). Genetic factors can range from risk factors of small effect size, which are common in the population, to rare variants that have a large effect size and, in some cases, can be considered causative.

Over the last 20 years, there has been great progress in the identification of common risk variants; for example, 287 common variants have been associated with schizophrenia (Trubetskoy *et al*., 2022). However, testing for these variants and calculating their combined effects using polygenic risk scoring does not (yet) have demonstrable clinical utility (Wray *et al*., 2021; Lewis and Vassos, 2022). Concurrently, there has been a rapid expansion in the catalogue of monogenic causes of intellectual disability (ID). A genomic diagnosis can now be made in approximately one-third of people with ID using WGS (Husson *et al*., 2020).

Several studies report that a significant proportion of people with neuropsychiatric disorders and their families would undergo genetic diagnostic testing if it were offered, with reported rates ranging from approximately 30–90%, depending on the condition, population studied, and the test’s purpose or predictive validity (Lawrence and Appelbaum, 2011; Cullen *et al*., 2022). Despite this, genetic testing is still uncommon in psychiatric practice (Soda *et al*., 2023). At the level of the individual and family, genetic diagnosis has many potential benefits, including: (1) helping patients and families understand their illness, (2) informing treatment and prognosis, (3) guiding screening and surveillance for associated mental and physical disorders, and (4) providing information about recurrence risk and enabling cascade testing. These potential benefits apply just as much to people living with neuropsychiatric disorders as those without. The identification of underlying genetic causes for neuropsychiatric disorders contributes to overall clinical care through precision medicine, targeted surveillance, empowerment, and reproductive choice (Kohler *et al*., 2017; Vorstman *et al*., 2017; Vorstman and Scherer, 2021). For some patients, understanding their genetic risk can also help reduce stigma by reframing neuropsychiatric disorders in biological terms rather than as a personal failing (Baek *et al*., 2023). Furthermore, genetic diagnoses can facilitate access to rare disease support groups and other community services for patients and their families (Manickam *et al*., 2021).

Studies on barriers to the implementation of genetic testing indicate that clinicians lack sufficient knowledge and experience in this area, emphasizing the importance of improving training and continuing education (Nurnberger *et al*., 2018). Many clinicians are unsure when genetic testing is indicated or how to interpret and communicate the results (Finn *et al*., 2005).

One important source of knowledge widely used by clinicians comprises clinical practice guidelines (CPGs). However, consistent CPGs for genetic testing in neuropsychiatric disorders appear to be lacking, with different countries and institutions having their own sets of guidelines. Moreover, CPGs are not always regularly updated, posing a risk of lagging behind the latest developments. Here, we aim to provide a comprehensive overview of the literature and CPGs concerning genetic testing in clinical practice for individuals with neuropsychiatric disorders in Europe. Our objective is to highlight both commonalities and differences, with the aim of enhancing understanding and reducing implementation barriers to recommendations in clinical settings.

## Methods

### Search strategy

A review of scientific databases (*PubMed*/*MEDLINE* and *Ovid*) was performed. Preferred Reporting Items for Systematic Reviews and Meta-Analysis (PRISMA) guidelines were adhered to as far as possible (Page *et al*., 2021). The search strategy focused on three primary concepts: genetic testing, psychiatry/mental disorders, and clinical practice guidelines. For each concept, keywords and medical subject headings were combined using the “OR” operator, and the results were combined using the “AND” operator (Supplementary Table 1, Supplemental digital content 1, https://links.lww.com/PG/A338). The search was restricted to English-language manuscripts published from 2010 onwards, based on the rationale that CPGs predating 2010 are unlikely to incorporate recent advancements in genetic testing.

In addition, websites from professional bodies from across Europe were screened to identify gray literature related to the research question. The focus was on European CPGs. The neuropsychiatric disorders specifically searched for included: autism spectrum disorder (ASD), dementia, psychosis, bipolar affective disorder, depressive disorder, Huntington’s disease (HD), attention deficit hyperactivity disorder (ADHD), personality disorders, anxiety, and eating disorders (Supplementary Table 1, Supplemental digital content 1, https://links.lww.com/PG/A338). These conditions were selected as they are common within mental health services and likely to be included in contemporary, region-specific clinical guidelines. Further data was retrieved through studying the reference lists of relevant CPGs. Our primary focus was on psychiatric guidelines, which might be of general clinical utility. However, we did not limit our search specifically to psychiatry-focused sources, recognizing the multidisciplinary relevance of genetic testing in neuropsychiatric disorders.

The initial database search was performed on the 11th of January 2024. All records were exported to EndNote 20 (2023 Clarivate) (The EndNote Team, 2013), where duplicates were removed using the “find duplicates” command, followed by manual verification. All titles and abstracts were screened, and the full-text manuscripts of potentially relevant clinical guidelines were retrieved for further analysis. All titles and abstracts retrieved were independently evaluated by two researchers (I.A. and M.H.) against inclusion and exclusion criteria. If consensus could not be reached, records were referred to a third and fourth reviewer (N.B. and J.Z.). The search was updated in October 2025; see Supplementary Table 1, Supplemental digital content 1, https://links.lww.com/PG/A338 for details.

### Inclusion and exclusion criteria

Included in the search were CPGs that included specific recommendations around genetic testing for neuropsychiatric disorders. CPGs developed by professional medical associations, government health agencies, healthcare institutions, international organizations, and independent expert panels, that were available online, were considered for the review. Only English-language CPGs from European countries were included in this review.

### Data extraction and analysis

A standardized data extraction table was developed to capture essential information from each CPG meeting inclusion criteria. Extracted data included authorship, title, year of publication, publishing organization, range of topics, target population (diagnosis and age), intended audience, and methodology/level of evidence. Data extraction was performed by one reviewer and independently verified by a second to ensure accuracy and consistency.

### Quality assessment

Eligible CPGs underwent basic appraisal by two reviewers (I.A. and M.H.) using the International Centre for Allied Health Evidence (iCAHE) Guideline Quality Checklist (Grimmer *et al*., 2014). The iCAHE is a validated tool designed to assess the quality of CPG development and methodology. The iCAHE checklist includes 14 items across six domains: availability, dates, supporting evidence, guideline developers, guideline purpose and users, and usability. Each item was scored as either 0 or 1, for a maximum score of 14. All CPGs, regardless of score, were included for review, with scores allowing for comparative analysis.

### Registration

PROSPERO registration was not possible, as reviews of CPGs do not meet their inclusion criteria.

## Results

### Study selection

**Fig. 1:**
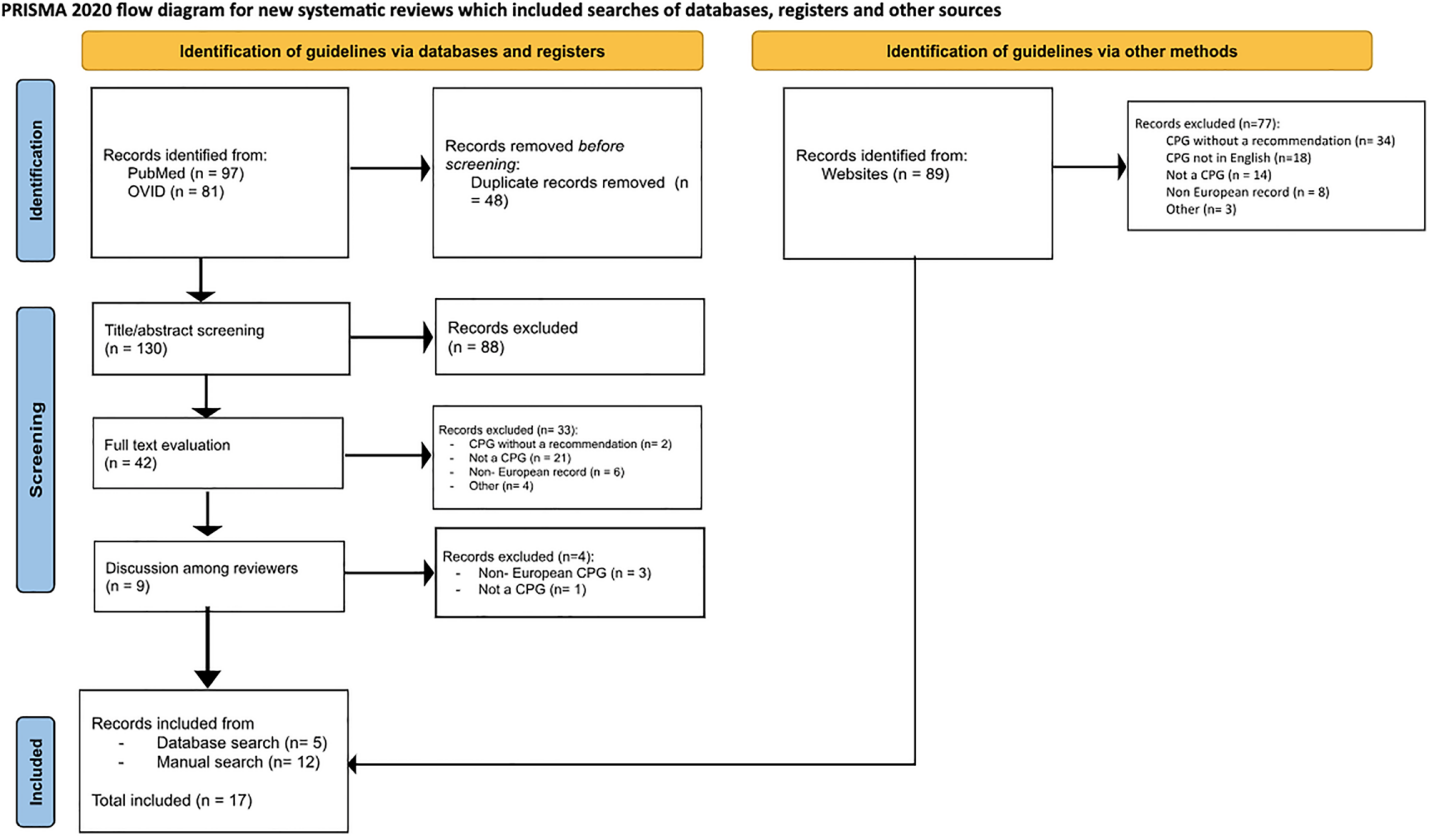
Flow diagram outlining search strategy.

A total of 267 records were identified from the database and manual website online searches. After deduplication, 130 records remained from the database search, and 89 records were identified through the manual search. There were various reasons for CPG exclusion during screening, including lack of recommendations on genetic testing, not being original CPGs, and being non-European (Fig. 1).

Following full-text evaluation, 17 CPGs were included in the review (Fig. 1). Of these, 12 were identified through the manual search and four through the first database search in December 2023. An additional CPG (published in May 2024) was identified during the database search update in October 2025, bringing the final total to 17 CPGs.

### General characteristics of the clinical practice guidelines

Of the 17 CPGs included in this review, six focused on neurodevelopmental disorders, 10 on dementia syndromes, and one provided recommendations for both neurodevelopmental conditions (including psychotic disorders) and dementia. The dementias covered include HD, Alzheimer’s dementia (AD), and frontotemporal lobar dementia (FTLD). The most recent CPG was published in May 2024. Eight CPGs were authored by Europe-wide institutions, five by institutions in England, one in Scotland, two in France, one in Ireland, and one in Switzerland. For ease of comparison, the CPGs were organized into two tables: Table 1 covers neurodevelopmental conditions, and Table 2 focuses on dementia syndromes. The Royal College of Psychiatrists’ College Report appears in both tables, as it includes recommendations for both categories (The Royal College of Psychiatrists, 2023).

**Table 1:** Table of results for clinical practice guidelines with recommendations for genetic testing in neurodevelopmental conditions

Publication year^a^	Guideline title	Reference	Country/countries	Diagnosis	Recommendation
2024	French guidelines for the diagnosis and management of Tourette syndrome	Hartmann *et al*., 2024	France	Tourette syndrome	No major gene involved in inheritance has been identified; therefore, neither molecular diagnosis nor presymptomatic diagnosis is possible in practice. Recommends that clinicians provide genetic counseling to explain the familial tendency and low absolute risk, providing reassurance to relatives of affected individuals.
2023	College report: The role of genetic testing in mental health settings	The Royal College of Psychiatrists, 2023	UK	ASD: children and adults	Routine genetic testing is not recommended for ASD; CNV testing is recommended in cases with co-occurring neurodevelopmental disorders, mental illness, congenital anomalies, or dysmorphic features. In the presence of an ID comorbidity, follow testing guidance for ID.
				ADHD in adults	Routine genetic testing in adults is not recommended. In the presence of an ID comorbidity, follow testing guidance for ID.
				ID: children and adults	ID: Fragile X testing, CMA, and whole genome sequencing are recommended as standard care for all children and adults. In adults, consider what has been tested/when, and how best to now support genetic diagnosis. Consider re-testing if needed.
				Schizophrenia	Schizophrenia: Test for CNVs in all young people with schizophrenia and in adults with co-occurring conditions (such as neurodevelopmental disorders, marked cognitive impairment, or congenital anomalies).
				Other indications:	Consider genetic testing in children/young people with: • Dysmorphic features; • Developmental delay/developmental intellectual disorder; • Unusual medical presentations^b^. Consider referral to local genetics service in familial clustering of neurodevelopmental or psychiatric disorders or epilepsy.
2021 (first published in 2012)	ASD spectrum disorder in adults: diagnosis and management	National Institute for Health and Care Excellence, 2012	UK	ASD (>18 years old)	Routine genetic testing is not recommended. Consider testing if there are specific dysmorphic features, congenital anomalies and/or evidence of a learning disability.
2021	ESCAP practice guidance for autism: a summary of evidence-based recommendations for diagnosis and treatment	Fuentes *et al*., 2021	Europe	ASD: children and adults	Basic assessment for all should include screening for pathogenic mutations in *MECP2*-gene and: • *PTEN*-gene analysis if concurrent macrocephaly; • Males: Fragile X testing; • Females: Fragile X if indicated by family history and/or phenotype.
2019 (first published in 2016)	Assessment, diagnosis and interventions for autism spectrum disorders	Scottish Intercollegiate Guidelines Network, 2016	Scotland	ASD: children and adults	Additional medical investigations, such as genetic testing, should be considered on an individual basis, taking into account the presence of dysmorphism and neurocutaneous stigmata, congenital anomalies, and intellectual disability or suspicion of epilepsy. Where clinically relevant, the need for chromosomal microarray and investigations to rule out recognized etiologies of ASD (e.g. tuberous sclerosis) should be reviewed for all individuals with ASD. Consultation with the local genetics service is advised for guidance on further testing.
2018	Autism spectrum disorder. Warning signs, detection, diagnosis and assessment in children and adolescents. Clinical practice guidelines method	Haute Autorité de Santé, 2018	France	ASD (<18 years old)	Routinely offer referral to medical geneticists, especially in ASD with any clinical sign suggestive of an underlying genetic disease (e.g. co-occurring with ID or a morphological abnormality) or if family genetic counseling is requested. If required, genetic testing will be arranged by these professionals.
2017 (first published 2011)	Autism spectrum disorder in under 19s: recognition, referral and diagnosis	National Institute for Health and Care Excellence, 2011	UK	ASD (<19 years old)	Consider genetic testing if specific dysmorphic features, congenital anomalies ± ID.

ADHD, attention deficit hyperactivity disorder; ASD, autism spectrum disorder; CMA, chromosomal microarray analysis; CNV, copy number variants; ESCAP, European Society for Child and Adolescent Psychiatry; ID, intellectual disability; *MECP2*, methyl-CpG-binding protein 2; *PTEN*, phosphatase and tensin homolog*.*

a Most recent version of guideline found.

b Unusual medical presentations include, for example, central hypopituitarism, skeletal anomalies, congenital heart conditions, epilepsy, neurocutaneous lesions, such as adenoma sebaceum, ash leaf macules, café-au-lait spots.

**Table 2:** Table of results for clinical practice guidelines with recommendations for genetic testing in dementia syndromes

Publication year^a^	Guideline title	Reference	Country/countries	Diagnosis	Recommendations
2023	College report: The role of genetic testing in mental health settings	The Royal College of Psychiatrists, 2023	UK	Dementia	Consider genetic investigations in all of those with: • Suspected frontotemporal dementia • Dementia with onset <55 years of age • Family history compatible with a dementia-causing genetic variant; • Clinical features suggestive of Down Syndrome (mosaic cases) or rare single-gene forms of dementia. Genetic testing for dementia risk variants (including *APOE4*) or polygenic risk scores is not recommended.
2023	Model of care for dementia in Ireland. Tullamore: National Dementia Services	Begley *et al*., 2023	Ireland	Dementia	Genetic testing in dementia should only be conducted at specialist memory clinics or memory assessment services with neurology expertise. Recommends referral for genetic screening and counseling (typically via a neurologist) in suspected young-onset dementia (<65 years old).
2018	Care and treatment of people with dementia, medical-ethical guidelines	Swiss Academy of Medical Sciences, 2018	Switzerland	Dementia	Genetic testing is not recommended for routine diagnostic assessment.
2018	Dementia: assessment, management and support for people living with dementia and their carers	National Institute for Health and Care Excellence, 2018	UK	Dementia	*APOE* genotyping is not recommended for routine diagnostic assessment.
2015	Diagnostic genetic testing for Huntington’s disease	Craufurd *et al*., 2015	Europe	Huntington’s disease	Diagnostic genetic testing is usually requested by neurologists, and is recommended for symptomatic individuals with symptoms that include unequivocal motor signs, rather than solely mood, personality, cognitive disturbance, or a family history without motor signs. Predictive testing can only be requested by genetic counseling units; therefore, individuals without motor symptoms who wish to know their genetic status will need referral to these services.
2013	EMQN/CMGS best practice guidelines for the molecular genetic testing of Huntington disease	Losekoot *et al*., 2013	Europe	Huntington’s disease	If HD is confirmed by DNA testing, the patient and family should be referred for genetic counseling and a possible offer of presymptomatic testing. Presymptomatic testing is recommended for individuals with an a priori risk of 50% or 25%, and may be considered for those with a 12.5% risk in rare cases. Presymptomatic testing is not recommended for individuals under 18 years old. However, minors with symptoms of juvenile HD may undergo diagnostic testing.
2013	Recommendations for the predictive genetic test in Huntington’s disease	MacLeod *et al*., 2013	Europe	Huntington’s disease	Predictive testing for HD is recommended only through a clinical geneticist, and the minimum recommended age is 18 years. Verify the family history of HD where possible. Minors at risk requesting the test should have access to genetic counseling and information.
2012	EFNS-ENS Guidelines on the diagnosis and management of disorders associated with dementia	Sorbi *et al*., 2012	Europe	Dementia syndromes outside of AD (including FTLD and HD)	Genetic screening for known pathogenic mutations should be conducted in specialist centers for individuals with relevant phenotypes or a family history of autosomal dominant dementia. Presymptomatic testing is recommended when there is a clear family history and a known mutation in an affected relative. It is recommended that Huntington’s disease protocol is followed.
2010	EFNS guidelines for the diagnosis and management of AD	Hort *et al*., 2010	Europe	AD	Routine *APOE* genotyping is not recommended Recommends screening for known pathogenic mutations in patients with an appropriate phenotype or a family history of an autosomal dominant dementia.
2010	EFNS guidelines on the molecular diagnosis of channelopathies, epilepsies, migraine, stroke, and dementias	Burgunder *et al*., 2010	Europe	Alzheimer’s dementia, early onset AD, and FTLD	For early-onset autosomal dominant AD, mutational screening is recommended in the sequence: *PSEN1, APP*, and if negative, *PSEN2*, to support genetic counseling. Consider genetic screening in sporadic cases of early-onset AD. Testing for *PGRN* and *MAPT* mutations is clearly indicated and useful for genetic counseling in autosomal dominant FTLD. Testing may also be considered in sporadic FTLD cases, although with a lower diagnostic yield If CADASIL is clinically suspected, direct sequencing of exons 3 and 4 of the Notch3 gene is recommended as an initial test.
2010 (first published in 2009)	EFNS guidelines on the molecular diagnosis of neurogenetic disorders: general issues, Huntington’s disease, Parkinson’s disease and dystonias	Harbo *et al*., 2009	Europe	Huntington’s disease	Recommends diagnostic testing for HD in patients with an otherwise unexplained clinical syndrome of a progressive choreatic movement disorder and neuropsychiatric disturbances with or without a positive family history of the disease. Testing for rare variants causing HD phenocopies is not recommended in mutation-negative cases.

AD, Alzheimer’s disease; *APOE*, Apolipoprotein E; *APP*, amyloid precursor protein; FTLD, frontotemporal lobar degeneration; HD, Huntington’s disease; *MAPT*, microtubule-associated protein tau; *PGRN*, progranulin; *PSEN1*, presenilin 1; *PSEN2*, presenilin 2; *PGRN*, progranulin.

a Most recent version of guideline found.

### Quality assessment

Each eligible CPG was assessed using the iCAHE guideline checklist, revealing significant variation in quality scores, which ranged from 7 to 14. The most common score was 12, achieved by 7 of the 17 guidelines (see Supplementary Table 2, Supplemental digital content 1, https://links.lww.com/PG/A338). While there is no definitive cutoff to classify CPGs as “high” or “low” quality, lower scores may indicate gaps in methodology, transparency, or usability, potentially reducing the guideline’s effectiveness.

### Neurodevelopmental disorders

Seven CPGs covering genetic testing in neurodevelopmental disorders were identified (National Institute for Health and Care Excellence, 2011, 2012; Scottish Intercollegiate Guidelines Network, 2016; Haute Autorité de Santé, 2018; Fuentes *et al*., 2021; The Royal College of Psychiatrists, 2023; Hartmann *et al*., 2024) (Table 1). Five of these CPGs specifically addressed ASD, with recommendations for testing provided by the European Society for Child and Adolescent Psychiatry (ESCAP), the Scottish Intercollegiate Guidelines Network, the French National Authority for Health (HAS), and the National Institute for Health and Care Excellence (NICE, for both children and adults). The sixth CPG from RCPsych offered broader recommendations, covering ASD, ADHD, ID, schizophrenia, and more general recommendations regarding genetic testing in clinical psychiatry. The seventh CPG was the only identified guideline for Tourette syndrome.

Among these, three CPGs covered ASD across all age groups (Scottish Intercollegiate Guidelines Network, 2016; Fuentes *et al*., 2021; The Royal College of Psychiatrists, 2023), while two focused on children (National Institute for Health and Care Excellence, 2011; Haute Autorité de Santé, 2018) and one specifically on adults (National Institute for Health and Care Excellence, 2012). ESCAP was the only CPG to recommend routine genetic testing, suggesting methyl-CpG-binding protein 2 (*MECP2*) mutation screening for all individuals, Fragile X testing for males (and for females where indicated), and phosphatase and tensin homolog (*PTEN*) analysis in cases of concurrent macrocephaly (Fuentes *et al*., 2021). The HAS guideline recommended routinely proposing a medical genetics consultation for children with ASD, particularly when clinical signs suggest a genetic etiology (Haute Autorité de Santé, 2018). Similarly, four CPGs advised genetic testing when clinically indicated, in the presence of features suggestive of an underlying genetic disease, such as co-occurring ID, dysmorphic features, or congenital anomalies (National Institute for Health and Care Excellence, 2011, 2012; Scottish Intercollegiate Guidelines Network, 2016; The Royal College of Psychiatrists, 2023).

Only the RCPsych guideline specifically addressed children and adults with ID (The Royal College of Psychiatrists, 2023). It recommends that Fragile X testing, chromosomal microarray analysis (CMA), and WGS should be made available for children and adults with ID as part of routine clinical care and that routine assessment pathways for genetic testing in all individuals with ID should be established.

Regarding ADHD, only the RCPsych guideline referred to adults with ADHD, stating that routine genetic testing for adults with ADHD without ID is not recommended (The Royal College of Psychiatrists, 2023). It also specified that familial clustering of neurodevelopmental disorders should be referred to local genetics services and that genetic testing should be considered in all children with dysmorphic features or developmental delay.

The recently published French CPG for the diagnosis and management of Tourette syndrome stated that while there is no role for genetic testing or a molecular diagnosis, the treating team should offer clear and appropriate genetic counseling to provide reassurance to families (Hartmann *et al*., 2024).

### Schizophrenia

The RCPsych guideline was the only source with explicit recommendations for genetic testing in schizophrenia (The Royal College of Psychiatrists, 2023). It suggested CNV testing for all children diagnosed with schizophrenia and advised that genetic testing be available to adults who present with additional features suggesting a genetic etiology, such as neurodevelopmental comorbidities, significant cognitive impairment, congenital abnormalities, or relevant family history.

### Dementia

Eleven CPGs referencing genetic testing in the context of dementia were retrieved (Harbo *et al*., 2009; Burgunder *et al*., 2010; Hort *et al*., 2010; Sorbi *et al*., 2012; Losekoot *et al*., 2013; MacLeod *et al*., 2013; Craufurd *et al*., 2015; National institute for health and care excellence, 2018; Swiss Academy of Medical Sciences, 2018; Begley *et al*., 2023; The Royal College of Psychiatrists, 2023) (Table 2). Genetic testing was the focus of six of these CPGs (Harbo *et al*., 2009; Burgunder *et al*., 2010; Losekoot *et al*., 2013; MacLeod *et al*., 2013; Craufurd *et al*., 2015; The Royal College of Psychiatrists, 2023). CPGs were provided by a range of bodies and specialties, underscoring the multidisciplinary nature of genetic testing guidelines for dementias in Europe.

Four CPGs on genetic testing in relation to HD were retrieved, three of which focused exclusively on HD. Recommendations concerning diagnostic and predictive testing were generally aligned. The European Federation of Neurological Societies (EFNS) 2010 and European Huntington’s Disease Network guideline (EHDN) 2015 guidelines (Harbo *et al*., 2009; Craufurd *et al*., 2015) recommend diagnostic testing based on phenotype, such as unequivocal motor signs. The European Molecular Genetics Quality Network/ Clinical Molecular Genetics Society (EMQN/CMGS) guideline recommends referring the patient and family for genetic counseling after a positive diagnostic test. It also states that presymptomatic predictive testing can be offered to adults with a 50% or 25% a priori risk of HD. The EHDN 2013 guideline recommends that presymptomatic testing should only be requested by a clinical geneticist, and both CPGs advise that it should only be offered to those under 18 in exceptional circumstances (MacLeod *et al*., 2013).

Outside of HD, no clear consensus on testing for suspected genetic forms of dementia emerged from the CPGs, with advice ranging from “genetic testing are not indicated for routine diagnostic testing” from the Swiss Academy of Medical Sciences (SAMS) guideline (Swiss Academy of Medical Sciences, 2018) to “if the clinical diagnosis is that of frontotemporal dementia, genetic testing for mutations in progranulin (*PGRN*) and microtubule-associated protein tau (*MAPT*) is clearly indicated” in the EFNS guideline from 2010 (Burgunder *et al*., 2010).

## Discussion

This review aimed to identify available CPGs relating to genetic testing in neuropsychiatric disorders and synthesize recommendations. While we identified both CPGs for dementia and for neurodevelopmental disorders, the majority were related to dementia, and the guidelines for neurodevelopmental disorders were more limited in numbers and scope. There were also notable gaps for psychiatric conditions such as schizophrenia. Furthermore, a substantial proportion of these CPGs were not from psychiatric bodies but from other medical specialities (e.g. neurology and clinical genetics) or from a health services perspective. Though areas of consensus were observed, recommendations varied widely. Below, we summarize findings and implications for research and clinical practice by diagnostic category. Please refer to Table 3 for examples of suitable genetic tests for some of the mentioned neuropsychiatric disorders.

**Table 3:** Examples of suitable genetic tests in genetic disorders

Type of genetic disorder	Example of a disorder	Type of genetic defect	Example of a suitable genetic test
Monogenetic The defect is in a single gene	Rett syndrome Cowden syndrome Huntington’s disease Fragile X syndrome Frontotemporal dementia	*MECP2* gene mutation *PTEN* gene mutation Repeat CAG in the *HTT* gene Repeat CGG in the *FMR1* gene *PGRN* gene mutation *MAPT* gene mutation	Whole Genome Sequencing (WGS) panel. *PTEN* Single gene sequencing. Single gene analysis or as part of a WGS panel. WGS based panel.
Copy number variants (CNVs) The defect may involve dozens of genes	22q11 deletion syndrome 16p11 duplication syndrome	Microdeletion of chromosome 22 Microduplication of chromosome 16	Microarray (array CGH or SNP array)
Chromosomal The defect may involve hundreds to thousands of genes	Down syndrome Klinefelter syndrome	Trisomy 21 47, XXY	Karyotyping

*FMR1*, fragile X messenger ribonucleoprotein 1; *HTT*, huntingtin; *MAPT*, microtubule-associated protein tau; *MECP2*, methyl-CpG-binding protein 2; *PGRN*, progranulin; *PTEN*, phosphatase and tensin homolog. Information on appropriate tests and what each test entails can be found across the following online resources:

• NHS England’s “National Genomic Test Directory: Testing Criteria for Rare and Inherited Disease (Version 8.1 – July 2025).” https://www.england.nhs.uk/wp-content/uploads/2018/08/rare-and-inherited-disease-eligibility-criteria-V8.1.pdf.

• Genomics England’s Catalogue of genes for WGS-based panels https://panelapp.genomicsengland.co.uk/panels/.

### Neurodevelopmental conditions

The RCPsych guideline was the only European CPG recommending genetic testing in children with developmental delay or ID, specifically advocating for Fragile X testing, CMA, and WGS as standard components of care (The Royal College of Psychiatrists, 2023). This aligns with American CPGs, where CMA and Fragile X testing are recommended as first-tier tests in cases without a specific syndrome identified (Manning and Hudgins, 2010; Michelson *et al*., 2011; Moeschler and Shevell, 2014; Muhle *et al*., 2017). The French guideline for the management of Tourette syndrome highlighted the importance of clear and appropriate genetic counseling (Hartmann *et al*., 2024).

Genetic causes are identifiable in up to 30–40% in individuals with both ASD and ID; in those with autism without ID, this percentage is much lower (between 0 and 8%) (Morris *et al*., 2021). In line with this, most European CPGs do not recommend routine genetic testing for ASD (or ADHD) unless additional clinical features are present, such as co-occurring ID or dysmorphic features. Although genetic causes are frequently linked to dysmorphic traits, this association is not universal, prompting recent literature to advocate for a low-threshold approach to genetic testing for all individuals with ASD (Srivastava *et al*., 2019). In alignment with this, the American College of Medical Genetics and Genomics has recommended Fragile X testing in all males and CMA testing as first-tier tests in the genetic evaluation of individuals with ASD for over a decade (Schaefer and Mendelsohn, 2013). The reasons for differences in recommendations across European and international CPGs remain unclear; they may relate to the speed at which research findings are translated into recommendations across the globe, perceived clinical utility, and cultural attitudes. However, such variation raises broader issues of equity in access to testing worldwide.

Variation may also reflect differences in the funding structures of national health systems. For example, European CPGs more often operate within publicly funded health systems and therefore emphasize cost-effectiveness and evidence thresholds, whereas recommendations from the USA evolve in the context of mixed public–private funding. This can allow faster adoption of emerging research and may also tolerate more expensive interventions (Cohen *et al*., 2013). However, issues in equity of access to testing are not so straightforward. Evidence from the USA shows that although genetic testing is widely recommended in ASD, financial and insurance-related barriers remain common reasons why families do not pursue testing, despite coverage by most public and private plans (Zhao *et al*., 2024). In contrast, international comparisons indicate a higher uptake of genetic testing for ASD in countries such as France, and it is thought that this could be associated with free access to healthcare (Amiet *et al*., 2014). These findings imply that equity concerns extend beyond variation in CPGs to also include the affordability and accessibility of testing for patients and families.

### Schizophrenia

The RCPsych guideline is the only CPG providing specific recommendations to test for rare copy number variants (CNVs) in schizophrenia with co-occurring features (The Royal College of Psychiatrists, 2023). Its recommendations are consistent with international CPGs from Canada, which recommend genetic testing in cases where ID or congenital abnormalities are also present (Addington *et al*., 2017).

Significant advances over the last decade have identified multiple rare, recurrent pathogenic CNVs, such as deletions in 22q11.2, 3q29, and 16p11.2, as clear risk factors for developing schizophrenia (Marshall *et al*., 2016). However, debate persists around the clinical utility of CNV testing for schizophrenia patients, given that these CNVs, while well-established risk factors, occur relatively infrequently (Morris *et al*., 2021).

The International Society of Psychiatric Genetics did not reach consensus on the widespread use of CNV testing in adult-onset disorders (International Society of Psychiatric Genetics (ISPG) 2019). However, it acknowledges that tests might have value in cases that present atypically or in the context of ID, ASD, and certain medical syndromes. It also emphasizes the importance of genetic counseling from clinicians with expertise in both mental health and genetics, with referral to a medical geneticist when results indicate a recognized genetic disorder or have wider health implications. In practice, we are aware of select centers where CNV testing is available for people with schizophrenia who have additional features suggesting a genetic etiology, for example, the All Wales Psychiatric Genomics Service (Kendall *et al*., 2024).

### Dementia

There is a good agreement across CPGs around diagnostic and predictive testing for HD. However, there was no clear consensus on testing for other suspected genetic forms of AD or FTLD. Interestingly, the one area of agreement was in relation to Apolipoprotein E (APOE) genotyping in AD. Three CPGs specifically counsel against routine *APOE* testing for diagnosing or predicting AD (Hort *et al*., 2010; National Institute for Health and Care Excellence, 2018; The Royal College of Psychiatrists, 2023).

The role of *APOE* testing in AD remains controversial, particularly given the rise of direct-to-consumer genetic testing, which often lacks the necessary pre- and post-test counseling. *APOE4* carriers, particularly homozygous individuals, are at substantially higher risk for AD, with emerging evidence suggesting that *APOE4* homozygosity may represent a distinct autosomal recessive form of the disease (Fortea *et al*., 2024). Additionally, pharmacogenetic testing for *APOE4* is becoming increasingly relevant, as it may be necessary to guide the use of new disease-modifying therapies like lecanemab, which carries heightened risks of amyloid-related imaging abnormalities for *APOE4* carriers (Cummings *et al*., 2023).

### Genomics, precision medicine, and psychiatry

Genomic data is fundamental to developing more personalized medicine. Health systems across Europe are in the process of extending genetic testing into mainstream services. For example, National Health Service (NHS) England has committed to becoming the “first national health care system to offer WGS as part of routine care” (NHS England, 2022). In most services, genetic testing is not yet part of routine psychiatric care. Psychiatric classification and practice encompass a wide range of disorders, making genetic testing in psychiatry a complex issue. Nonetheless, there are some disorders and areas of psychiatric practice where genetic testing has clear relevance. 22q11.2 deletion syndrome and Fragile X syndrome are two genomic diagnoses with particular relevance to psychiatry.

Fragile X syndrome has a strong association with developmental and psychiatric symptoms; it is the most prevalent inherited cause of mild-to-severe ID and the most common monogenic cause of ASD (Kidd *et al*., 2014). The detection of Fragile X can offer families valuable information about risk to family members and to future children, giving those affected agency, a practical understanding of the condition, and enabling early diagnosis (Raspa *et al*., 2016). Genetic diagnosis further allows clinicians to anticipate and manage significant associated medical comorbidities, such as mitral valve prolapse, otitis media, and seizures, which is crucial for a subgroup of patients often disproportionately affected by health disparities (Lozano *et al*., 2016).

Deletion of chromosome 22q11.2 is associated with an elevated risk of seizures, cardiac abnormalities, and adult-onset psychosis, particularly within the schizophrenia spectrum (Bassett and Chow, 2008; Schneider *et al*., 2014). Recognizing this association enables clinicians to closely monitor symptomatology and initiate timely, tailored treatment when required. Furthermore, evidence suggests that these patients may benefit from lower doses of antipsychotics, preventive antiepileptic treatment, and blood electrolyte monitoring (Heslop *et al*., 2014; Fung *et al*., 2015). Both examples underscore the clinical importance of genetic testing for certain individuals with mental illness, enabling more precise care and supporting efforts to address disparities in mental health outcomes.

The advent of massively parallel sequencing, commonly known as next-generation sequencing (NGS), has revolutionized DNA sequencing (Wheeler *et al*., 2008). WGS – the rapid and cost-effective sequencing of an individual’s entire 3 billion base pair DNA – is increasingly being utilized in clinical genetics as a first-line investigation. For example, NHS England Genomic Medicine Service, now offers a WGS neurodegeneration virtual panel that screens for variants across 120 genes associated with neurodegenerative diseases (Genomics England, 2024). Additionally, private healthcare providers are expanding access to WGS, as demonstrated by Bupa’s recent pilot program offering WGS to selected UK customers (Bupa Newsroom, 2024). However, this is a relatively recent development, and with the possible exception of the RCPsych report, the CPGs identified here predate this paradigm shift. The variation in CPGs may, in part, have a temporal explanation. The pace of change in genetic testing would seem to necessitate regular updating of CPGs.

While this review focuses on diagnostic genetic testing, it is important to acknowledge the growing role of pharmacogenomic testing in psychiatry. Pharmacogenomics applies knowledge of genetic variation to guide medication choice and dosing, and several relevant genes influencing psychiatric drug metabolism have been identified. Of particular relevance are allelic variations in the genes encoding the CYP2D6 and CYP2C19 enzymes, which are involved in the metabolism of antidepressants and antipsychotics. A recent large-scale study of preemptive genome-guided treatment in psychiatry reported multiple benefits among individuals with actionable phenotypes for these enzymes, including a reduced risk of adverse reactions, fewer re-admissions, and lower treatment costs when compared with treatment as usual (Skokou *et al*., 2024).

The International Society of Psychiatric Genetics notes that while evidence for broad pharmacogenetic testing in psychiatry remains limited, available CYP2C19 and CYP2D6 results should be incorporated into prescribing decisions, particularly for patients with previous adverse reactions or inadequate treatment response. However, human leukocyte antigen (HLA)-A and HLA-B testing before use of carbamazepine and oxcarbazepine is currently recommended to avoid serious side effects (e.g. Stevens-Johnson syndrome) (International Society of Psychiatric Genetics (ISPG) 2019).

CPGs for pharmacogenomics-guided prescribing are currently available from the Dutch Pharmacogenomics Working Group (KNMP, 2024) and the Clinical Pharmacogenetics Implementation Consortium (CPIC). In the UK, the Centre for Excellence in Regulatory Science and Innovation in Pharmacogenomics is currently working on guidelines for pharmacogenomic testing within the NHS (University of Liverpool, 2025). The emerging evidence and ongoing CPG development highlight the potential of pharmacogenomics to significantly influence psychiatric practice in the near future.

### Barriers to genetic testing

Implementing genetic testing recommendations in clinical settings remains a significant challenge, with testing prevalence often falling short of guidelines. For example, despite established recommendations, fewer than 5% of children with ASD are referred for genetic testing (Adlington *et al*., 2019; Moreno-De-Luca *et al*., 2020). Barriers include limited awareness among mental health professionals, logistical issues such as the lack of clinical pathways linking mental health and genomic medicine services (Pinzón-Espinosa *et al*., 2022), and insufficient clinician expertise in this area. A survey of UK child and adolescent psychiatrists highlighted this gap, revealing a preference to refer patients to regional genetics services over directly ordering genetic tests (Nurnberger *et al*., 2018; Wolfe *et al*., 2018).

Furthermore, obtaining informed consent for genetic testing can be complex, particularly for patients with developmental delays who may require tailored communication approaches and family involvement (Bang *et al*., 2022). Therefore, enhancing clinician training, education, and confidence in genetic testing is essential to integrate these practices into psychiatric care effectively (Nurnberger *et al*., 2018).

Additionally, it has been proposed that patients can benefit from genetic counseling in itself to explain the contribution of genetic factors to the development of neuropsychiatric disorders and help manage uncertainty. Ideally, these conversations should be initiated by the patient’s own psychiatrist and be guided by the patient’s understanding of genetic contributions to mental illness (Peay and Claire Austin, 2011). In addition, genetic counseling can be helpful when initially discussing genetic testing, to help patients and their relatives to better understand their diagnosis and the implication (Eeltink *et al*., 2021). Without this, there can be mixed experiences with regard to genetic testing (Strnadová *et al*., 2023).

Finally, documented ethnic disparities in ASD diagnosis reveal that children of color, particularly those with co-occurring ID, often experience delayed or missed ASD diagnoses (Mandell *et al*., 2009). Similar inequities have also been observed in access to genetic screening and counseling (Shea *et al*., 2013). A recent UK study on perceptions of genetic testing found that ethnic minority individuals are less likely than white individuals to have taken or heard about genetic testing and are more apprehensive about its impact on employability (Harris *et al*., 2023). These findings highlight the need to consider patient education and ensure equitable access to genetic counseling when developing new initiatives, aiming to prevent the widening of existing health disparities.

### Working with clinical genetics

In addition to recommendations for specific disorders, the RCPsych report advocates for developing testing pathways in collaboration with genomic medicine services (The Royal College of Psychiatrists, 2023). Close liaison between psychiatric services, clinical geneticists, genetic counselors, and clinical scientists will become increasingly important as more complex WGS results are returned. The optimal structure and process for such coordination will vary depending on the healthcare system and available services. However, a multidisciplinary team model, which evaluates the appropriateness of testing and interprets genetic variants, is widely employed and considered best practice.

### The future

It seems likely that long-read sequencing will become the predominant sequencing technology, as it allows simultaneous detection of single-nucleotide variants (SNVs) and CNVs (Logsdon *et al*., 2020). This has significant implications for the investigation of neurodevelopmental disorders, with a much increased single-test yield anticipated. For example, microarray has a CNV yield of around 10% when used for the investigation of developmental delay/ID. It is estimated that the combined CNV and SNV yield will be in the region of 30% for the same indication (Lindstrand *et al*., 2019).

In the limited number of centers where testing for people with schizophrenia is being undertaken, investigation is primarily by microarray. However, recent exome sequencing studies have identified at least 10 genes where ultra-rare coding variants confer a substantial increased risk for schizophrenia with odds ratios in the range of the schizophrenia risk CNVs (Singh *et al*., 2022). The ability of long-read sequencing to detect both CNVs and SNVs, and therefore potentially identify both classes of variant, could make it invaluable for comprehensive testing in complex mental disorders.

Genome-wide association studies have shown that many mental disorders are polygenic. For example, major depression is highly polygenic with at least 308 high-confidence gene associations (Adams *et al*., 2025). Individually associated variants have a small impact on risk. However, the polygenic risk scoring method allows the effect of multiple risk variants to be summated for an individual (Purcell *et al*., 2009). While polygenic risk scores have influenced our understanding of and guided research into the biological etiology of many mental disorders, at present, they lack sufficient predictive power for use in clinical practice.

The availability of appropriate genetic counseling will be key to the offer of genetic investigation for more genetically complex and heterogeneous disorders, such as schizophrenia. Evidence is emerging that multidisciplinary clinics bringing together genetic counseling, medical genetics, and psychiatric expertise can deliver genetic investigation safely and effectively in this context (Kendall *et al*., 2024). Adoption of such a multidisciplinary model is advocated.

Additionally, a multiprofessional survey across Europe indicated that a lack of CPGs is a major barrier to the implementation of genetic investigation in psychiatry (Koido *et al*., 2023). Development of an inclusive European network of clinical psychiatric geneticists would facilitate the understanding of current practice, exchange of knowledge, and implementation of CPGs.

### Strengths and Limitations

The primary strength of this review is its broad scope, enhanced by an extensive online search alongside a regular database search. Additionally, categorizing findings for ease of use provides a practical reference for clinicians, facilitating CPG accessibility in diverse psychiatric contexts. Limitations of the review are its focus on European, English-language CPGs, which may restrict international applicability and may omit relevant recommendations from non-English-speaking regions.

Furthermore, we conducted both database and manual searches to try to identify all relevant, available guidelines. Additional CPGs were returned in the manual searching. This was not unexpected, as CPGs are not always published in scientific literature, although they are based on research findings and papers. While this potentially limited reproducibility, we aimed to mitigate the risk of major CPGs being missed by ensuring a structured online search strategy, conducted by two independent reviewers.

Also, CPGs not available online may have been excluded, potentially limiting the comprehensiveness of the results. Pharmacogenomic testing recommendations were excluded, narrowing the scope; however, this was deemed beyond the focus of the current review.

### Conclusion

This review highlights the heterogeneity in recommendations, reflecting perhaps the slower integration of genetic testing within mental health compared with other medical specialties. Based on our findings, we recommend considering genetic testing for patients with ID and for those with ASD who exhibit features suggestive of a genetic cause, such as ID and/or dysmorphic characteristics (in the case of ASD). In these situations, genetic testing may help clarify diagnoses, identify syndromic causes, and guide personalized treatment. Careful consideration of clinical utility, patient autonomy, and access to genetic counseling are essential to provide informed and supportive care.

## Acknowledgements

### Conflicts of interest

There are no conflicts of interest.

## Supplementary Material
