# Supplementary material for: What do European clinical guidelines say about genetic testing for people with neuropsychiatric disorders? A scoping review
Source: Psychiatr Genet. 2025 Dec 3;36(1):1–12. doi: 10.1097/YPG.0000000000000407 (PMC12834271; doi:10.1097/YPG.0000000000000407)
Supplement: Supplementary file 1 [file pg-36-01-s001.docx]

Supplementary Table 1. Search strategy.

|  | Search details: |
| --- | --- |
| Database Search | Initial search date: December 2023  Search update: 28^th^ October 2025  Pubmed:  ("guide*"[Title] OR "recommend*"[Title]) AND ("test*"[Title/Abstract] OR "screen*"[Title/Abstract]) AND ("mental disorders"[MeSH Major Topic] OR "psychiatr*"[Title/Abstract]) AND ("genetic*"[Title/Abstract] OR "genom*"[Title/Abstract]) AND ((english[Filter]) AND (2010:2023[pdat]))  Results = 97  OVID search:  Ovid MEDLINE(R) ALL <1946 to October 28, 2025>  APA PsycInfo <1806 to October 2025 Week 3>  Econlit <1886 to October 23, 2025>    1             "guide*".m_titl.              295047  2             "recommend*".m_titl. 90014  3             mental disorders.mp. [mp=ti, ab, tx, ct, bt, ot, nm, hw, fx, kf, ox, px, rx, ui, sy, ux, mx, tc, id, tm, mf]                479887  4             psychiatr*.mp. [mp=ti, ab, tx, ct, bt, ot, nm, hw, fx, kf, ox, px, rx, ui, sy, ux, mx, tc, id, tm, mf]                1009867  5             1 or 2    378522  6             3 or 4    1252147  7             test*.ti. or test*.ab. or screen*.ab. or screen*.ti.         6814760  8             genet*.ti. or genet*.ab. or genom*.ab. or genom*.ti.  2254353  9             5 and 6 and 7 and 8       109  10          9 and 2010:2025.(sa_year).      90  11          limit 10 to english language      81 |
| Manual search | Date: December 2023- Feb 2024  Google search for: ‘(X), (Y), guideline’  (X)= Mental health conditions including: Autism spectrum disorder, Dementia, Psychosis, Bipolar affective disorder, Depressive disorder, Developmental delay, Intellectual disability, Huntington’s, ADHD, Personality disorders, Anxiety and Eating disorders.  (Y)= European countries including France, Germany, Spain, Italy, Netherlands, Finland, Sweden, Turkey, Belgium, Switzerland, Denmark, Estonia, Latvia, Lithuania, Austria, Ireland, Albania, Bosnia and Herzegovina, Bulgaria, Croatia, Hungary, Czech Republic, Hungary, Poland, Romania, Serbia, Greece, Malta.  *The Google search was conducted using combinations of a named mental disorder, a European country, and the term “guideline.” This produced approximately 320 search combinations. The Google search was conducted independently by two researchers (i.e. each researcher conducted the ~320 searches). Results were compared and a final list produced.* |

Supplementary Table 2. International Centre for Allied Health Evidence (iCAHE) Guideline Quality Checklist scores of each included clinical guideline.

| **Guideline Title** | **Reference** | **1** | **2** | **3** | **4** | **5** | **6** | **7** | **8** | **9** | **10** | **11** | **12** | **13** | **14** | **Total /14** |
| --- | --- | --- | --- | --- | --- | --- | --- | --- | --- | --- | --- | --- | --- | --- | --- | --- |
| French guidelines for the diagnosis and management of Tourette syndrome | (Hartmann et al. 2024) | 1 | 1 | 0 | 1 | 1 | 0 | 0 | 0 | 0 | 0 | 1 | 1 | 1 | 1 | 8 |
| College Report: The role of genetic testing in mental health settings | (The Royal College of Psychiatrists 2023) | 1 | 1 | 1 | 1 | 0 | 0 | 0 | 0 | 0 | 0 | 1 | 1 | 1 | 1 | 8 |
| Model of care for dementia in Ireland | (Begley et al. 2023) | 1 | 1 | 1 | 1 | 0 | 1 | 0 | 0 | 0 | 0 | 1 | 1 | 1 | 1 | 9 |
| Care and treatment of people with dementia, medical-ethical guidelines | (Swiss Academy of Medical Sciences 2018) | 1 | 1 | 0 | 1 | 0 | 0 | 0 | 0 | 0 | 0 | 1 | 1 | 1 | 1 | 7 |
| Dementia: assessment, management and support for people living with dementia and their carers | (National institute for health and care excellence 2018) | 1 | 1 | 0 | 1 | 0 | 1 | 1 | 1 | 1 | 1 | 1 | 1 | 1 | 1 | 12 |
| Diagnostic genetic testing for Huntington's disease | (Craufurd et al. 2015) | 1 | 1 | 1 | 0 | 0 | 0 | 0 | 0 | 0 | 0 | 1 | 1 | 1 | 1 | 7 |
| EMQN/CMGS best practice guidelines for the molecular genetic testing of Huntington disease | (Losekoot et al. 2013) | 1 | 1 | 0 | 1 | 0 | 0 | 0 | 0 | 0 | 0 | 1 | 1 | 1 | 1 | 7 |
| Recommendations for the predictive genetic test in Huntington's disease | (MacLeod et al. 2013) | 1 | 1 | 1 | 1 | 1 | 0 | 1 | 0 | 0 | 0 | 1 | 1 | 1 | 1 | 10 |
| EFNS-ENS Guidelines on the diagnosis and management of disorders associated with dementia | (Sorbi et al. 2012) | 1 | 1 | 1 | 1 | 0 | 0 | 1 | 1 | 1 | 1 | 1 | 1 | 1 | 1 | 12 |
| EFNS guidelines for the diagnosis and management of Alzheimer's disease | (Hort et al. 2010) | 1 | 1 | 1 | 1 | 0 | 0 | 1 | 1 | 1 | 1 | 1 | 1 | 1 | 1 | 12 |
| EFNS guidelines on the molecular diagnosis of channelopathies, epilepsies, migraine, stroke, and dementias | (Burgunder et al. 2010) | 1 | 1 | 1 | 1 | 0 | 0 | 1 | 1 | 1 | 1 | 1 | 1 | 1 | 1 | 12 |
| EFNS guidelines on the molecular diagnosis of neurogenetic disorders: general issues, Huntington’s disease, Parkinson’s disease and dystonias | (Harbo et al. 2009) | 1 | 1 | 1 | 1 | 0 | 0 | 1 | 1 | 1 | 1 | 1 | 1 | 1 | 1 | 12 |
| Autism spectrum disorder in adults: diagnosis and management | (National Institute for Health and Care Excellence 2012) | 1 | 1 | 1 | 1 | 0 | 0 | 1 | 1 | 1 | 1 | 1 | 1 | 1 | 1 | 12 |
| ESCAP practice guidance for autism: a summary of evidence-based recommendations for diagnosis and treatment | (Fuentes et al. 2021) | 1 | 1 | 1 | 1 | 0 | 1 | 1 | 0 | 0 | 0 | 1 | 1 | 1 | 1 | 10 |
| Assessment, diagnosis and interventions for autism spectrum disorders | (Scottish Intercollegiate Guidelines Network 2016) | 1 | 1 | 1 | 1 | 1 | 1 | 1 | 1 | 1 | 1 | 1 | 1 | 1 | 1 | 14 |
| Autism spectrum disorder: Warning signs, detection, diagnosis and assessment in children and adolescents. Clinical practice guidelines method. | (Haute Autorité de Santé 2018) | 1 | 1 | 1 | 1 | 1 | 1 | 1 | 0 | 0 | 0 | 1 | 1 | 1 | 1 | 11 |
| Autism spectrum disorder in under 19s: recognition, referral and diagnosis | (National Institute for Health and Care Excellence 2011) | 1 | 1 | 1 | 1 | 0 | 0 | 1 | 1 | 1 | 1 | 1 | 1 | 1 | 1 | 12 |

Q 1-3: Availability, Q4- 6: Dates, Q7-10: Underlying evidence, Q11- 12: Guideline developers, Q13: Guideline purpose and users and Q14: Ease of use.
